# Partial Replacement of Animal Fat with Full-Fat Almond in Broiler Chicken Diets: Performance, Nutrient Digestibility, Blood Profile, Cecal-Fecal Microflora Composition, and Foot-Pad Dermatitis

**DOI:** 10.3390/ani11113075

**Published:** 2021-10-28

**Authors:** Yong Ju Kim, Min Ho Song, Ji Hwan Lee, Han Jin Oh, Se Yeon Chang, Jae Woo An, Young Bin Go, Dong Cheol Song, Hyun Ah Cho, Byoung Kon Lee, Seok Hyeon Cho, Hyeun Bum Kim, Jin Ho Cho

**Affiliations:** 1 Department of Animal Sciences, Chungbuk National University, Cheongju 286-44, Korea; xormakzm@naver.com (Y.J.K.); junenet123@naver.com (J.H.L.); dhgkswls17@naver.com (H.J.O.); angella2425@naver.com (S.Y.C.); blueswing547@naver.com (J.W.A.); rhdudqls3@gmail.com (Y.B.G.); paul741@daum.net (D.C.S.); hannah0928@naver.com (H.A.C.); bklee0418@cherrybro.com (B.K.L.); 2 Division of Animal and Dairy Science, Chungnam National University, Daejeon 341-34, Korea; mhsong@cnu.ac.kr; 3 Pathway Intermediates Inc., Seoul 062-53, Korea; danny.cho@pathway-intermediates.com; 4 Department of Animal Resource, and Science, Dankook University, Cheonan 311-16, Korea

**Keywords:** full-fat almond, fat alternative, growth performance, nutrient digestibility

## Abstract

**Simple Summary:**

Recently, as the price of grains in feed increases worldwide, interest in new raw materials that can replace protein and fat sources is increasing. This study evaluated the effect of full-fat almonds (FFA) on broiler nutrition. In the study, the formulation of FFA as a raw material showed higher growth performance than the basic feed. This seems to be due to the high digestibility of protein and fat and the promotion of intestinal health through the reduction of harmful microorganisms in the intestine. Therefore, FFA can be used as a high-quality protein and fat substitute as a raw material for broiler feed.

**Abstract:**

The purpose of this study was to evaluate the effect of full-fat almonds (FFA) as an alternative protein and fat source for broiler feed on broiler productivity, nutrient digestibility, blood characteristics, cecal-fecal microflora, and foot-pad dermatitis (FPD). A total of 96, one-day-old broiler chickens (Arbor Acres) with initial body weight 41.61 ± 0.36 g were placed in 16 cages. In each trial, four treatments were set up: a basal diet partially replacing animal fat with FFA 0% (Control, CON), a basal diet partially replacing animal fat with FFA 1% (T1), a basal diet partial replacing animal fat with FFA 2% (T2), a basal diet partially replacing animal fat with FFA 4% (T3). The experiment was conducted for a total of 4 weeks. Feed conversion ratio (FCR) was higher (*p* < 0.05) in the T3 group of broilers at weeks 0 to 1 than in the CON group of broilers. From weeks 3 to 4, and for the entire experimental period, FCR was lower (*p* < 0.05) in the T3 group of broilers than in the CON and T1 groups of broilers. The apparent ileal digestibility (AID) of the ether extract (EE) was higher (*p* < 0.05) in the T3 group than in the other treatment groups, and AID of crude protein (CP) was higher (*p* < 0.05) in the T3 group than in the CON group. The apparent total tract digestibility (ATTD) of EE was lower (*p* < 0.05) in the CON group than in the other treatment groups, and the ATTD of CP and energy was higher (*p* < 0.05) in the T3 group of broilers than in the CON group of broilers. The AID and ATTD of total amino acids were higher (*p* < 0.05) in the T3 group than in the other treatment groups. Blood cholesterol levels were lower (*p* < 0.05) in the T3 group of broilers than in the CON and T1 groups of broilers, and higher (*p* < 0.05) in the CON group of broilers than in the T2 and T3 groups of broilers. The amount of *E. coli* in the cecal and fecal was lower (*p* < 0.05) in the T3 group than in CON and T1 groups. FPD score was higher (*p* < 0.05) in the T3 group of broilers than in the CON group of broilers. In conclusion, replacing a partial of animal fat with at least 4% FFA in broiler diets can increase growth performance and nutrient digestibility in broiler nutrition.

## 1. Introduction

Poultry farming is one of the most accelerated and technological fields in global agriculture [1]. Advances in genetics, nutrition, health, and management technology have resulted in highly efficient and organized poultry farming that can produce low-cost, high-biologically valuable animal protein for human consumption [2]. Poultry feeding is one of the important sectors of the poultry industry as it accounts for 70–80% of the total farming cost [3]. Changes in the price of raw materials used for feed production have a great impact on the income of poultry farms. In the case of corn and soybean meal, which are raw materials typically used for poultry formulations, international grain prices have been continuously rising because of weather changes, and prices fluctuate frequently due to external factors such as exchange rate fluctuations. For this reason, interest in raw materials suitable for poultry feed with relatively low prices and small fluctuations is increasing.

Almonds (*Prunus dulcis*) are attracting attention as a raw material to replace these unstable feed ingredients. The United States accounts for 80% of the world’s almond production, and California is the main producer. Over the past decade, US almond production has steadily increased from 640,000 t to 900,000 t per year [4]. Almonds contain lipids (about 50%), proteins (about 20%), and carbohydrates (about 20%), have low water content, and contain a variety of hydrophobic bioactive compounds [5]. Almonds have a nutritionally desirable fatty acid profile, with oleic (65%) and linoleic (25%) acids accounting for approximately 90% of the total almond lipids [6]. Moreover, the fiber and polyphenol content of almonds serves as a substrate for intestinal microbial fermentation, contributing to the regulation of the microflora composition [7,8]. At present, data on the effect of full-fat almonds as a poultry feed ingredient are limited. Therefore, the purpose of this study was to determine the effect of full-fat almonds as a feed ingredient on growth performance, nutrient digestibility, blood profiles, cecal-fecal microflora composition, and foot-pad dermatitis in broiler chickens.

## 2. Materials and Methods

### 2.1. Ethics

The experimental protocol was approved (CBNUA-1531-21-02) by the Institutional Animal Care and Use Committee of Chungbuk National University, Cheongju, Korea.

### 2.2. Preparation and Analysis of Full-Fat Almonds

Full-fat almonds (FFA) are manufactured by Blue Diamond Growers Company, California, USA and sourced from Easybio Company, Seoul, Korea. Four samples of FFA were prepared and analyzed for dry matter (DM), crude protein (CP), ether extract (EE), crude fiber (CF), ash, calcium (Ca), and phosphorus (P) according to AOAC [9]. The results are shown in Table 1.

### 2.3. Animals and Housing

A total of 96, one-day-old broiler chickens (Arbor Acres, Arbor Acres broilers were obtained from a local hatchery (Cherrybro CO., Asan, Korea)) with initial body weight 41.61 ± 0.36 g were placed in 16 cages (100 cm width, 40 cm depth, and 45 cm height). Each cage (3 male and 3 female chicks/cage) was equipped with two nipple drinkers connected to a common water line supply. The experiment was conducted for a total of 4 weeks, lighting schedule was 23 L:1 D at 100 lx on day 1, 12 L:12 D at 30 lx on day 4 until week 2, and 8 L:16 D at 30 lx thereafter. The experiment initiation temperature was 33 ± 1 °C, and the temperature was lowered by 2 °C every week to maintain 24 °C at the end of the experiment.

### 2.4. Dietary Treatments

The 16 cages were randomly assigned to 4 dietary treatments with 4 replicates of 6 broiler chickens each: basal diet partial replacing animal fat with FFA 0% (Control, CON), basal diet partial replacing animal fat with FFA 1% (T1), basal diet partial replacing animal fat with FFA 2% (T2), basal diet partial replacing animal fat with FFA 4% (T3). The base of the diet was formulated according to growth stage requirements (FFA 1% replaces 15% of animal fat, Table 2, Table 3, Table 4 and Table 5). All diets were formulated to meet or exceed NRC [10] nutrient requirements for poultry. The birds had free access to the diet and water throughout the experimental period.

### 2.5. Growth Performance

Body weight (BW), body weight gain (BWG), feed intake (FI), and feed conversion ratio (FCR) were recorded on d 7, 14, 21 and 28. All birds within a pen were weighed at each time point (6 birds were sampled in each cage, with 4 replicates per treatment). The BW gain was calculated as the BW of the current time point subtracted the BW of the previous time point. Feed intake was calculated by subtracting the remaining feed amount from the initial feed amount, and FCR was calculated by dividing FI by BWG.

### 2.6. Nutrient Digestibility

Broiler chickens were euthanized by cervical dislocation. Determination of the apparent digestibility of nutrients was performed by collecting both total and ileal digesta. All experimental diets were mixed with 0.2% Cr_2_O_3_ before collecting digesta. The ileal digesta was collected from Meckel’s diverticulum 1 cm before the ileal-cecal junction. The rectum digesta was collected from rectum 3 cm before the cloaca to collect fecal without urine. The digesta were gently squeezed, rinsed with saline, and collected in a plastic pillbox. All feed, ileal sample, and rectum sample were finely ground after drying for 72 h at 50 °C oven and analyzed for DM, CP, energy, amino acid (AA), and EE. The energy was determined using a calorimeter (model 1261, Parr Instrument Company, Moline, IL, USA). Analyses of DM, CP and EE were made according to the methodology described in AOAC [9] and analysis of AA made in High Performance Liquid Chromatography (HPLC) (SHIMADZU, Model LC-10AT, Shimadzu Corp., Kyoto, Japan) methodology [11,12]. The apparent total tract digestibility (ATTD) and apparent ileal digestibility (AID) percentage were calculated using the following equation: AID% = 100 − [100 × (% Cr_2_O_3_ in diet/% Cr_2_O_3_ in ileal digesta) × (% nutrient in ileal digesta/% nutrient in diet)], ATTD% = 100 − [100 × (Cr_2_O_3_ in diet/Cr_2_O_3_ in rectum digesta) × (nutrient in rectum digesta/nutrient in diet)].

### 2.7. Blood Profiles

On day 28, blood samples (2 mL) were collected from the wing vein of broiler chickens (one broiler chicken randomly chosen from each cage), and serum was harvested and stored in a deep freeze at −20 °C for further analysis. Concentrations of glucose, total protein, blood urea nitrogen, creatinine, and total cholesterol were determined colorimetrically using a UV-visual spectrophotometer (Microlab 200: Merck Laboratory Analyzer, New Delhi, India) using commercial kits (Prism diagnostic Pvt. Ltd., Mumbai, India).

### 2.8. Cecal-Fecal Microflora Composition

On day 28, cecal samples were collected (one broiler chicken randomly chosen from each cage) immediately in sterile glass containers after slaughter. Fecal samples were collected for each treatment group before the end of the experiment, and then immediately analyzed. After homogenization by suspending in aseptic distilled water, samples were used for measuring the number of viable cells by serial dilution from 10^−3^ to 10^−7^. Bacterial colonies were counted by the pour plate method. In order to measure the number of *Salmonella*, *Lactobacillus* and *E. coli*, Salmonella-Shigella agar for *Salmonella*, MRS agar for *Lactobacillus*, and MacConkey agar for *E. coli* were used, and *Salmonella*, *E. coli* was cultured at 37 °C. for 24 h, and *Lactobacillus* was cultured for 48 h.

### 2.9. Foot-Pad Dermatitis (FPD)

The incidence and severity of FPD were measured on day 28 using the scoring method described by Eichner [13]: no lesion (score 0), lesion covering less than 25% of the sole of the foot (score 1), large area lesion, covering between 25% and 50% of the sole of the foot (score 2), more than 50% of the lesion of the plantar (score 3). Scoring was performed by three independent observers for all birds in the trial. Assessments were performed on both paws. The average score was used for statistical analysis.

### 2.10. Statistical Analysis

Data collected during the study were subjected to analysis of variance (ANOVA) for Completely Randomized Design [14] using General Linear Model Procedure (SAS, 2010). The statistical model used to test the effects of treatment on growth performance, nutrient digestibility, blood profiles, cecal-fecal microflora composition, FPD is presented as follows: Yij = μ + Pi + Eij. Where: Yij = Observed value of a dependent variable; μ = Overall mean; Pi = Effect of different levels of FFA; and Eij = Residual error. The differences between means were tested for significance (*p* < 0.05) using the LSD range test.

## 3. Results

### 3.1. Growth Performance

The growth performance data are shown in Table 6. BW was higher (*p* < 0.05) in the T3 group of broilers at weeks 2 and weeks 4 than in the CON group of broilers. From weeks 1 to 2, weeks 3 to 4, and for the entire experimental period, BWG was higher (*p* < 0.05) in the T3 group of broilers than in the CON group of broilers. From weeks 0 to 1, FI was higher (*p* < 0.05) in the T3 group of broilers than in the CON group of broilers. From weeks 3 to 4, FI was higher (*p* < 0.05) in the T1 group of broilers than in the T3 group of broilers. FCR was higher (*p* < 0.05) in the T3 group of broilers at weeks 0 to 1 than in the CON group of broilers. From weeks 3 to 4, and for the entire experimental period, FCR was lower (*p* < 0.05) in the T3 group of broilers than in the CON and T1 groups of broilers.

### 3.2. Nutrient Digestibility

The AID data are shown in Table 7. The AID of the EE was higher (*p* < 0.05) in the T3 group of broilers than in the other treatment groups of broilers. The AID of CP was higher (*p* < 0.05) in the T3 group of broilers than in the CON group of broilers. The AID of histidine and valine were higher (*p* < 0.05) in the T3 group of broilers than in the CON and T1 groups of broilers. The AID of leucine was higher (*p* < 0.05) in the T3 group of broilers than in the CON group of broilers. The AID of threonine was higher (*p* < 0.05) in the CON group of broilers than in the T2 group of broilers. The AID of methionine was higher (*p* < 0.05) in the CON and T3 groups of broilers than for the other treatment groups of broilers. The AID of lysine and total essential AAs were higher (*p* < 0.05) in the T3 group of broilers than in the other treatment groups of broilers. The AID of aspartic acid was higher (*p* < 0.05) in the T3 group of broilers than in the T1 group of broilers. The AID of serine was higher (*p* < 0.05) in the T3 group of broilers than in the other treatment groups of broilers, and lowest (*p* < 0.05) in the T2 group of broilers. The AID of the total non-essential AAs and total AAs was higher (*p* < 0.05) in the T3 group of broilers than in the other treatment groups of broilers.

The ATTD data are shown in Table 8. The ATTD of EE was lower (*p* < 0.05) in the CON group of broilers than in the other treatment groups of broilers. The ATTD of CP and energy was higher (*p* < 0.05) in the T3 group of broilers than in the CON group of broilers. The ATTD of arginine, histidine, and isoleucine was higher (*p* < 0.05) in the T3 group of broilers than in the other treatment groups of broilers, and lowest (*p* < 0.05) in the CON group of broilers. The ATTD of methionine was higher (*p* < 0.05) in the CON and T3 groups of broilers than in the other treatment groups of broilers. The ATTD of valine was higher (*p* < 0.05) in the T3 group of broilers than in the CON and T1 groups of broilers. The ATTD of lysine, glycine, and the total essential AAs was higher (*p* < 0.05) in the T3 group of broilers than in the other treatment groups of broilers. The ATTD of alanine, proline, and tyrosine was higher (*p* < 0.05) in the T3 group of broilers than in the CON and T2 groups of broilers. The ATTD of serine was higher (*p* < 0.05) in the T1 and T3 groups of broilers than in the other treatment groups of broilers. The ATTD of aspartic acid and glutamic acid were higher (*p* < 0.05) in the T3 group of broilers than in the other treatment groups of broilers. The ATTD of total non-essential AAs and total AAs was higher (*p* < 0.05) in the T3 group of broilers than in the other treatment groups of broilers.

### 3.3. Blood Profiles

The blood profile data are shown in Table 9. Blood cholesterol levels were lower (*p* < 0.05) in the T3 group of broilers than in the CON and T1 groups of broilers, and higher (*p* < 0.05) in the CON group of broilers than in the T2 and T3 groups of broilers.

### 3.4. Cecal-Fecal Microflora Composition

The cecal-fecal microflora composition data are shown in Table 10. The amount of *E. coli* in the cecal was lower (*p* < 0.05) in the T3 group of broilers than in the CON and T1 groups of broilers, and higher (*p* < 0.05) in the CON group of broilers than in the T2 and T3 groups of broilers. The amount of *E. coli* in the fecal was lower (*p* < 0.05) in the T3 group of broilers than in the other treatment groups of broilers.

### 3.5. Foot-Pad Dermatitis

The FPD data are shown in Table 11. The FPD score was higher (*p* < 0.05) in the T3 group of broilers than in the CON group of broilers.

## 4. Discussion

### 4.1. Growth Performance

The chickens fed diets with FFA up to 4% showed negative FCR results compared to those in the control group at phase 1 (0–1 week). However, the FCR in phase 4 (3–4 weeks) and the entire experimental period showed positive results in chickens fed a diet containing 4% FFA compared to the CON group. There is no report in the literature regarding the effect of FFA on the performance of broiler chickens. Consistent with our results, Oliveira [15] reported that the dietary inclusion of some components, such as sorghum, may increase feed conversion rates in broiler chicks due to their tannin content. This increase may also be related to the effect of phytates on the availability of mineral elements, and the negative effect of condensed tannins, which are known to affect the FI of animals by inhibiting the activity of digestive enzymes and reducing the absorptive capacity of the feed [16]. Therefore, the increase in FCR in the chickens in phase1 was possibly due to the anti-nutritional effect of the FFA fiber. However, Awad et al. [17] found that birds younger than 2 weeks had more proteobacteria, which increase pro-inflammatory cytokines, whereas Firmicutes and Tenericutes, which increase anti-inflammatory cytokines were predominant in birds older than 2 weeks. These results indicate that with the growth of the chicken’s digestive tract, fiber can increase the intestinal health and digestibility. In this experiment, the FCR of chickens treated with 4% almond mixture was high in phase 1 and low in phase 4 and overall experimental period. Annongu et al. [18] showed that treated supplementation of enzyme on fermented almond fruit waste improved the FI, BWG, and FCR of cockerels. Freitas et al. [19] also reported that an increase in cashew nut bran in broiler diets promoted a linear increase in BWG and FCR for the whole experiment period. There is a close relationship between gut health, nutrition, and microflora. The gut microbiota is a highly metabolic organ that consumes about 20% of dietary energy [20]. Many soluble fibers act as prebiotics when present in the feed, directly promoting the growth of beneficial gut bacteria and short-chain fatty acid (SCFA) production [21,22,23]. Similarly, insoluble fiber also potentially affects the colonization of beneficial gut microbiota [24]. Including certain types of insoluble fiber, such as 3–5% cellulose in the diet, has been proven to improve nutrient utilization. Dietary fiber (DF) also increases pancreatic enzyme activity and inverse peristalsis, which leads to increased nutrient digestibility [25,26,27]. Reverse peristalsis allows the bile salts to reach the gizzard and mix with gastric secretions to improve fat emulsification, reducing the likelihood that fat will coat nutrients and consequently, making them more readily hydrolyzed and absorbed [28].

### 4.2. Nutrient Digestibility

In our study, the AID and ATTD of the EE, CP, and amino acids in chickens fed diets with up to 4% FFA showed higher digestibility compared to the CON group of broilers. Published data on the energy, EE, CP, and amino acid digestibility of FFA for poultry are scarce. However, the higher digestibility of EE was due to monounsaturated acids and polyunsaturated unsaturated acids. Özcan et al. [29] reported that the major fatty acids of almond kernel oil were oleic (72.5–79.9%), linoleic (13.5–19.8%), and palmitic acids (5.9–6.7%). Previous studies have reported that saturated fats (rich in long-chain fatty acids) were less digestible than fats rich in unsaturated fatty acids [30,31]. In addition, the degree of fat saturation affects inverse peristalsis and the endogenous loss of fat, so unsaturated fats generate more bile lobes and enzymes in the intestinal lumen compared to saturated fats, thereby prolonging the exposure time of the digestive contents to digestive enzymes [32]. After emulsification with conjugated bile salts, polyunsaturated fatty acids (PUFAs) in dietary fat are hydrolyzed by pancreatic lipase to a mixture consisting essentially of 2-monoacylglycerides and free fatty acids. The binding of monoglycerides with bile salts combined with long-chain unsaturated fatty acids immediately forms micelles [33]. Micelles play an important role in solubilizing low-polarity fatty acids and fat-soluble vitamins and inducing absorption through the intestinal epithelium [34].

The increase in digestibility of CP and AA in this experiment was thought to be due to the changes in fiber content. Several studies have reported increased nutrient digestibility in chickens fed diets with structural components such as fiber [35,36]. The presence of structural components, coarse particles, and fiber in chicken feed increased gizzard activity and residence time above the gastrointestinal tract (GIT). It increased bacterial fermentation in crops [37] and decreased intestinal pH [38], thereby improving pepsin activity and thus, increasing protein digestibility [39]. In addition, lower pH as a result of higher SCFAs produced by beneficial bacteria in the gut may enhance pepsin activity [40], which has been reported to increase the denaturation and hydrolysis of dietary proteins. However, it is necessary to elucidate the exact mechanism through additional studies in the future.

### 4.3. Blood Profiles

Nuts are known to be nutritious food with high lipid content. In our experiment, the T2 and T3 treatment groups with 2% and 4% FFA content in feed showed significantly lower blood cholesterol content compared to the FFA 0% treatment group. Almonds increase high-density lipoprotein (HDL) cholesterol and reduce low-density lipoprotein (LDL) cholesterol levels in humans when included in the diet [41]. A previous study reported that including almonds in the diet reduced plasma cholesterol, triglycerides (TG), and LDL cholesterol levels, and increased HDL cholesterol levels in broilers [42]. Similarly, Arjomandi et al. [43] found that blood cholesterol levels in quails fed diets containing 20% and 30% almonds were significantly lower than those of quails fed diets containing 10% almonds and the controls. They also reported that quails fed diets containing 20% and 30% almonds had significantly lower LDL cholesterol levels than quails fed a control diet. Three major factors that affect blood cholesterol levels following almond intake are monounsaturated fatty acids (MUFAs) and PUFAs, vitamin E, and fiber [44,45,46]. A diet rich in MUFA and PUFA has been reported to reduce plasma cholesterol levels in birds and humans [47,48]. Vitamin E has been reported to significantly inhibit cholesterol biosynthesis by regulating the gene encoding a key enzyme in the cholesterol biosynthesis pathway [49]. It has been reported that dietary fiber intake reduced LDL cholesterol in the blood by reducing cholesterol absorption, increasing bile acid synthesis, and producing SCFAs that inhibit cholesterol synthesis [50,51].

### 4.4. Cecal-Fecal Microflora Composition

In this experiment, the 4% FFA content in the feed significantly reduced the *E. coli* content in the cecal and fecal compared to other treatments. Almonds contain significant amounts of indigestible carbohydrates that can be used as potential prebiotics. Prebiotics are non-digestible feed ingredients that can serve as a substrate for microbes, help shape the gut microbiome, and develop immune capacity [52,53]. Mandalari et al. [7,54] investigated the prebiotic effect of almond seeds and almond brown skins using a mixed fecal bacterial culture in vitro and found that almond seeds and almond brown skins significantly increased the population of bifido-bacteria after gastric and duodenal simulated digestion in vitro. Other polysaccharides, such as NSP-β-1,3/1,6 glucans have been reported to reduce the number of *E. coli* in the intestine by binding glucans to glucan receptors on leukocytes [55].

### 4.5. Foot-Pad Dermatitis

Nutrition is a very important factor in broiler rearing as it affects drinking water intake, manure viscosity, and litter quality. Dietary factors that increase the amount of water consumed by chickens thin the manure and increase the litter moisture content, which, in turn, leads to the development of FPD [56]. There are no studies on the effect of almonds as a feed ingredient on FPD in broilers. In this study, broilers fed a diet with 4% almonds showed significantly higher FPD scores compared to those fed basal diets. These results appear to be due to the non-starch polysaccharides (NSP) and high mineral content of almonds. Dunlop et al. [57] reported feed ingredients high in NSP, such as wheat, barley, and rye, retained moisture and prevented the reabsorption of moisture in the intestine, resulting in watery and viscous broiler manure. In addition, when minerals such as sodium or potassium were high, the amount of water consumed was increased and the litter moisture content was increased [58].

## 5. Conclusions

In summary, the present study revealed that the partial replacement of animal fat with low levels (1–2%) of full-fat almonds in the broiler diets during the overall experimental period (week 0–4) had no positive effect compared to the control group. Contrastively, 60% animal fat replaced with 4% full-fat almond in broiler diets showed higher growth performance, nutrient digestibility, and lower blood cholesterol, content of *E. coli* in cecal-fecal microflora compared to the control group. The results of this study indicate that 60% animal fat replacement with at least 4% full-fat almond in broiler diets can increase growth performance and nutrient digestibility.

## Figures and Tables

**Table 1:** Chemical composition on full-fat almond (%).

DM	CP	EE	CF	Ash	Ca	P
92.4	19.0	34.4	30.4	3.6	0.4	0.4

Abbreviations: DM, dry matter; CP, crude protein; EE, ether extract; CF, crude fiber; Ca, calcium; P, phosphorus.

**Table 2:** Ingredient composition of experimental diets (phase 1/day 1–7).

Ingredients (%)	Level of Sweet Almond (%)			
	0 (CON)	1 (T1)	2 (T2)	4 (T3)
Corn	37.2	37.0	37.0	36.1
Wheat fine	15.3	15.2	15.0	15.0
Rice pollards	2.4	2.4	2.4	2.4
Soybean oil meal	26.9	26.8	26.5	26.2
Cookie wheat flour	1.9	2.0	2.0	2.0
Almond, Toasted	0.0	1.0	2.0	4.0
DDGS	5.0	4.9	4.9	4.9
Tankage meat meal	2.8	2.8	2.7	2.5
Meat-bone meal	2.5	2.5	2.4	2.3
Poultry offal meal	1.0	1.0	1.0	1.0
Feather meal	0.0	0.0	0.0	0.0
Animal fat	1.7	1.5	1.2	0.7
L-lysine	0.6	0.6	0.6	0.6
L-methionine	0.4	0.4	0.4	0.4
L-threonine	0.2	0.2	0.2	0.2
L-tryptophan	0.1	0.1	0.1	0.1
Salt	0.2	0.2	0.2	0.2
Limestone	0.5	0.5	0.5	0.5
MDCP	0.2	0.2	0.2	0.2
Liquid-Choline	0.1	0.1	0.1	0.1
Vitamin premix ^a^	0.3	0.3	0.3	0.3
Mineral premix ^b^	0.3	0.3	0.3	0.3
Chemical composition (%)				
Crude protein	23.3	23.2	23.2	23.2
Ether extract	5.5	5.5	5.5	5.6
Crude fiber	3.4	3.7	3.7	3.8
Crude ash	5.8	5.8	5.9	5.9
Calcium	0.9	0.9	0.9	0.9
Phosphorus	0.5	0.5	0.5	0.5
Lysine	1.5	1.5	1.5	1.5
SAA	1.1	1.1	1.1	1.1
AMEn(kcal/kg)	3000	3000	3000	3000

Abbreviations: CON, basal diet; T1, partial replacing animal fat with full-fat almonds 1%; T2, partial replacing animal fat with full-fat almonds 2%; T3, partial replacing animal fat with full-fat almonds 4%; DDGS, Dried distiller’s grains with soluble; MCDP, Mono-dicalcium phosphate; SAA, sulfur amino acids; AMEn, nitrogen-corrected apparent metabolizable energy. ^a^ Supplied per kilogram diet: vitamin A (retinyl acetate), 9000 IU; vitamin D3, 3000 IU; vitamin E (DL-α-tocopheryl acetate), 48 mg; vitamin K, 3 mg; thiamin, 1.8 mg; riboflavin, 6 mg; pyridoxine, 3 mg; vitamin B12, 0.012 mg; niacin, 42 mg; folic acid, 1.2 mg; biotin, 0.24 mg; pantothenic acid, 12 mg. ^b^ Supplied per kilogram of diet: manganese, 120 mg; zinc, 100 mg; iron, 80 mg; copper, 20 mg; iodine, 2 mg; selenium, 0.3 mg; cobalt, 0.5 mg.

**Table 3:** Ingredient composition of experimental diets (phase 2/day 8–14).

Ingredients (%)	Level of Sweet Almond (%)			
	0 (CON)	1 (T1)	2 (T2)	4 (T3)
Corn	41.6	41.5	41.3	41.0
Wheat fine	15.1	15.1	15.0	15.0
Rice pollards	2.5	2.5	2.5	2.5
Soybean oil meal	21.0	20.5	20.8	20.8
Cookie wheat flour	2.0	2.0	2.0	1.0
Almond, Toasted	0.0	1.0	2.0	4.0
DDGS	7.0	7.0	7.0	6.9
Tankage meat meal	0.5	0.5	0.5	0.5
Meat-bone meal	3.0	3.0	2.0	2.3
Poultry offal meal	2.0	2.0	2.0	1.5
Feather meal	0.6	0.6	0.6	0.6
Animal fat	1.9	1.6	1.3	0.8
L-lysine	0.6	0.6	0.6	0.6
L-methionine	0.3	0.3	0.3	0.4
L-threonine	0.1	0.1	0.1	0.1
L-tryptophan	0.1	0.1	0.1	0.1
Salt	0.2	0.2	0.2	0.2
Limestone	0.6	0.5	0.5	0.5
MDCP	0.2	0.2	0.5	0.5
Liquid-Choline	0.1	0.1	0.1	0.1
Vitamin premix ^a^	0.3	0.3	0.3	0.3
Mineral premix ^b^	0.3	0.3	0.3	0.3
Chemical composition (%)				
Crude protein	21.3	21.2	21.2	21.2
Ether extract	5.9	6.0	6.0	6.1
Crude fiber	3.4	3.6	3.6	4.0
Crude ash	5.3	5.4	5.3	5.4
Calcium	0.8	0.8	0.8	0.8
Phosphorus	0.6	0.6	0.6	0.6
Lysine	1.3	1.3	1.3	1.3
SAA	1.0	1.0	1.0	1.0
AMEn(kcal/kg)	3020	3020	3020	3020

Abbreviations: CON, basal diet; T1, partial replacing animal fat with full-fat almonds 1%; T2, partial replacing animal fat with full-fat almonds 2%; T3, partial replacing animal fat with full-fat almonds 4%; DDGS, Dried distiller’s grains with soluble; MCDP, Mono-dicalcium phosphate; SAA, sulfur amino acids AMEn, nitrogen-corrected apparent metabolizable energy. ^a^ Supplied per kilogram diet: vitamin A (retinyl acetate), 9000 IU; vitamin D3, 3000 IU; vitamin E (DL-α-tocopheryl acetate), 48 mg; vitamin K, 3 mg; thiamin, 1.8 mg; riboflavin, 6 mg; pyridoxine, 3 mg; vitamin B12, 0.012 mg; niacin, 42 mg; folic acid, 1.2 mg; biotin, 0.24 mg; pantothenic acid, 12 mg. ^b^ Supplied per kilogram of diet: manganese, 120 mg; zinc, 100 mg; iron, 80 mg; copper, 20 mg; iodine, 2 mg; selenium, 0.3 mg; cobalt, 0.5 mg.

**Table 4:** Ingredient composition of experimental diets (phase 3/day 15–21).

Ingredients (%)	Level of Sweet Almond (%)			
	0 (CON)	1 (T1)	2 (T2)	4 (T3)
Corn	45.2	45.1	45.1	44.8
Wheat fine	15.6	15.0	15.3	15.0
Rice pollards	2.5	2.4	2.5	2.4
Soybean oil meal	17.7	17.8	17.1	17.2
Cookie wheat flour	2.0	2.0	2.0	1.8
Almond, Toasted	0.0	1.0	2.0	4.0
DDGS	6.0	6.0	6.0	5.9
Tankage meat meal	1.5	1.4	0.9	1.0
Meat-bone meal	2.0	2.1	2.2	1.8
Poultry offal meal	2.0	2.0	2.0	1.8
Feather meal	0.9	0.9	0.9	0.8
Animal fat	1.9	1.6	1.3	0.8
L-lysine	0.6	0.6	0.6	0.3
L-methionine	0.3	0.3	0.3	0.4
L-threonine	0.1	0.1	0.1	0.1
L-tryptophan	0.1	0.1	0.1	0.1
Salt	0.2	0.2	0.2	0.2
Limestone	0.5	0.5	0.5	0.5
MDCP	0.2	0.2	0.2	0.4
Liquid-Choline	0.1	0.1	0.1	0.1
Vitamin premix ^a^	0.3	0.3	0.3	0.3
Mineral premix ^b^	0.3	0.3	0.3	0.3
Chemical composition (%)				
Crude protein	20.2	20.1	20.1	20.1
Ether extract	6.0	6.1	6.1	6.2
Crude fiber	3.2	3.4	3.6	4.0
Crude ash	5.1	5.1	5.0	5.1
Calcium	0.8	0.8	0.8	0.8
Phosphorus	0.5	0.5	0.5	0.5
Lysine	1.2	1.2	1.2	1.2
SAA	1.0	1.0	1.0	1.0
AMEn(kcal/kg)	3070	3070	3070	3070

Abbreviations: CON, basal diet; T1, partial replacing animal fat with full-fat almonds 1%; T2, partial replacing animal fat with full-fat almonds 2%; T3, partial replacing animal fat with full-fat almonds 4%; DDGS, Dried distiller’s grains with soluble; MCDP, Mono-dicalcium phosphate; SAA, sulfur amino acids; AMEn, nitrogen-corrected apparent metabolizable energy. ^a^ Supplied per kilogram diet: vitamin A (retinyl acetate), 9000 IU; vitamin D3, 3000 IU; vitamin E (DL-α-tocopheryl acetate), 48 mg; vitamin K, 3 mg; thiamin, 1.8 mg; riboflavin, 6 mg; pyridoxine, 3 mg; vitamin B12, 0.012 mg; niacin, 42 mg; folic acid, 1.2 mg; biotin, 0.24 mg; pantothenic acid, 12 mg. ^b^ Supplied per kilogram of diet: manganese, 120 mg; zinc, 100 mg; iron, 80 mg; copper, 20 mg; iodine, 2 mg; selenium, 0.3 mg; cobalt, 0.5 mg.

**Table 5:** Ingredient composition of experimental diets (phase 4/day 22–28).

Ingredients (%)	Level of Sweet Almond (%)			
	0 (CON)	1 (T1)	2 (T2)	4 (T3)
Corn	48.9	48.7	48.4	48.1
Wheat fine	15.2	15.1	15.2	15.0
Rice pollards	2.6	2.6	2.5	2.4
Soybean oil meal	15.5	15.2	15.3	15.0
Cookie wheat flour	2.0	2.0	2.0	1.8
Almond, Toasted	0.0	1.0	2.0	4.0
DDGS	5.0	5.0	5.0	4.9
Tankage meat meal	1.7	1.5	1.2	1.1
Meat-bone meal	1.5	1.6	1.5	1.1
Poultry offal meal	2.2	2.2	2.1	2.0
Feather meal	0.8	0.8	0.8	0.8
Animal fat	1.9	1.6	1.3	0.8
L-lysine	0.5	0.5	0.5	0.5
L-methionine	0.4	0.4	0.4	0.4
L-threonine	0.1	0.1	0.1	0.1
L-tryptophan	0.1	0.1	0.1	0.1
Salt	0.2	0.2	0.2	0.2
Limestone	0.5	0.5	0.5	0.5
MDCP	0.2	0.2	0.2	0.5
Liquid-Choline	0.1	0.1	0.1	0.1
Vitamin premix ^a^	0.3	0.3	0.3	0.3
Mineral premix ^b^	0.3	0.3	0.3	0.3
Chemical composition (%)				
Crude protein	19.1	19.1	19.0	19.0
Ether extract	5.8	5.9	5.9	5.9
Crude fiber	3.0	3.3	3.5	3.8
Crude ash	4.8	4.8	4.8	5.0
Calcium	0.7	0.7	0.7	0.7
Phosphorus	0.5	0.5	0.5	0.5
Lysine	1.1	1.1	1.1	1.1
SAA	1.0	1.0	1.0	1.0
AMEn(kcal/kg)	3100	3100	3100	3100

Abbreviations: CON, basal diet; T1, partial replacing animal fat with full-fat almonds 1%; T2, partial replacing animal fat with full-fat almonds 2%; T3, partial replacing animal fat with full-fat almonds 4%; DDGS, Dried distiller’s grains with soluble; MCDP, Mono-dicalcium phosphate; SAA, sulfur amino acids; AMEn, nitrogen-corrected apparent metabolizable energy. ^a^ Supplied per kilogram diet: vitamin A (retinyl acetate), 9000 IU; vitamin D3, 3000 IU; vitamin E (DL-α-tocopheryl acetate), 48 mg; vitamin K, 3 mg; thiamin, 1.8 mg; riboflavin, 6 mg; pyridoxine, 3 mg; vitamin B12, 0.012 mg; niacin, 42 mg; folic acid, 1.2 mg; biotin, 0.24 mg; pantothenic acid, 12 mg. ^b^ Supplied per kilogram of diet: manganese, 120 mg; zinc, 100 mg; iron, 80 mg; copper, 20 mg; iodine, 2 mg; selenium, 0.3 mg; cobalt, 0.5 mg.

**Table 6:** Effects of full-fat almonds on growth performance in broiler chickens.

Items	CON	T1	T2	T3	SEM	*p*-Value
BW (g)						
Initial BW	42	42	42	42	0	1.000
1 W	168	174	173	170	2	0.685
2 W	394 ^b^	416 ^ab^	415 ^ab^	424 ^a^	4	0.016
3 W	970	982	989	988	9	0.898
4 W	1566 ^b^	1623 ^ab^	1623 ^ab^	1684 ^a^	15	0.039
**BWG (g)**						
0–1 W	126	132	131	128	2	0.667
1–2 W	226 ^b^	242 ^ab^	242 ^ab^	254 ^a^	3	0.030
2–3 W	576	566	574	564	6	0.952
3–4 W	596 ^b^	641 ^ab^	634 ^ab^	696 ^a^	11	0.013
0–4 W	1524 ^b^	1581 ^ab^	1581 ^ab^	1642 ^a^	14	0.038
**FI (g)**						
0–1 W	127 ^c^	140 ^b^	141 ^ab^	147 ^a^	2	0.001
1–2 W	313	308	310	325	5	0.694
2–3 W	867	870	858	873	9	0.956
3–4 W	1161 ^ab^	1200 ^a^	1100 ^ab^	1084 ^b^	17	0.035
0–4 W	2467	2518	2409	2429	21	0.300
**FCR**						
0–1 W	1.008 ^b^	1.061 ^ab^	1.076 ^ab^	1.148 ^a^	0.019	0.049
1–2 W	1.385	1.273	1.281	1.280	0.018	0.300
2–3 W	1.505	1.537	1.495	1.548	0.013	0.396
3–4 W	1.948 ^a^	1.872 ^a^	1.735 ^ab^	1.557 ^b^	0.043	0.003
0–4 W	1.619 ^a^	1.593 ^a^	1.524 ^ab^	1.479 ^b^	0.014	0.003

Abbreviations: CON, basal diet; T1, partial replacing animal fat with full-fat almonds 1%; T2, partial replacing animal fat with full-fat almonds 2%; T3, partial replacing animal fat with full-fat almonds 4%; BW, body weight; BWG, body weight gain; FI, feed intake; FCR, feed conversion ratio; SEM, standard error of means. Each value is the mean value of 4 replicates (6 broiler/cage). ^a–^^c^ Means within column with different superscripts differ significantly (*p* < 0.05).

**Table 7:** Effects of full-fat almonds on AID of nutrient and amino acids in 28 days broiler chickens.

Items (%)	CON	T1	T2	T3	SEM	*p*-Value
Apparent ileal digestibility						
Dry matter	71.60	71.80	69.70	70.13	0.42	0.217
Ether extract	77.37 ^b^	77.61 ^b^	77.96 ^b^	80.69 ^a^	0.43	0.004
Crude protein	67.92 ^b^	70.56 ^ab^	71.52 ^ab^	73.71 ^a^	0.66	0.004
Energy	67.23	68.35	68.63	70.44	0.49	0.096
**Essential amino acids**						
Arginine	72.02	71.36	71.32	76.59	0.91	0.110
Histidine	60.60 ^b^	62.63 ^b^	67.18 ^ab^	71.90 ^a^	1.43	0.008
Isoleucine	61.16 ^b^	62.93 ^b^	73.50 ^a^	73.70 ^a^	1.78	0.001
Leucine	68.79 ^ab^	68.21 ^b^	71.57 ^ab^	78.08 ^a^	1.42	0.033
Lysine	72.58 ^b^	71.58 ^b^	73.52 ^b^	81.19 ^a^	1.15	0.001
Methionine	79.15 ^a^	64.57 ^b^	62.08 ^b^	77.19 ^a^	2.03	0.001
Phenylalanine	70.10	70.62	70.77	72.25	0.79	0.828
Threonine	67.61 ^a^	64.61 ^ab^	60.09 ^b^	66.45 ^ab^	1.01	0.022
Valine	63.07 ^b^	66.87 ^b^	69.52 ^ab^	74.12 ^a^	1.29	0.005
Tryptophan	57.30	67.22	66.13	67.01	1.86	0.176
Glycine	63.77	66.14	64.01	69.07	0.83	0.065
Total	69.05 ^b^	67.90 ^b^	69.80 ^b^	75.17 ^a^	0.88	0.003
**Non-essential amino acids**						
Alanine	68.22	66.69	62.79	71.40	1.35	0.148
Aspartic acid	70.31 ^ab^	65.98 ^b^	70.33 ^ab^	73.85 ^a^	0.96	0.016
Cysteine	61.44	59.90	59.36	62.25	1.06	0.792
Glutamic acid	72.02 ^ab^	69.93 ^b^	68.65 ^b^	74.95 ^a^	0.74	0.002
Proline	62.12	61.89	61.80	67.57	0.89	0.035
Serine	70.07 ^b^	67.14 ^bc^	62.46 ^c^	77.11 ^a^	1.55	0.001
Tyrosine	63.08	64.32	62.26	67.04	1.09	0.472
Total	68.21 ^b^	66.63 ^b^	65.82 ^b^	71.11 ^a^	0.42	0.002
Total amino acids	68.58 ^b^	67.18 ^b^	67.60 ^b^	69.21 ^a^	0.78	0.002

Abbreviations: CON, basal diet; T1, partial replacing animal fat with full-fat almonds 1%; T2, partial replacing animal fat with full-fat almonds 2%; T3, partial replacing animal fat with full-fat almonds 4%; SEM, standard error of means. Each value is the mean value of 4 replicates. ^a–c^ Means within column with different superscripts differ significantly (*p* < 0.05).

**Table 8:** Effects of full-fat almonds on ATTD of nutrient and amino acids in 28 days broiler chickens.

Items (%)	CON	T1	T2	T3	SEM	*p*-Value
Apparent total tract digestibility						
Dry matter	77.25	78.77	77.08	79.55	0.41	0.069
Ether extract	84.95 ^b^	86.98 ^a^	86.15 ^a^	86.97 ^a^	0.31	0.041
Crude protein	73.53 ^b^	75.60 ^ab^	75.92 ^ab^	76.64 ^a^	0.42	0.034
Energy	74.59 ^b^	77.24 ^ab^	77.08 ^ab^	78.03 ^a^	0.48	0.045
**Essential amino acids**						
Arginine	80.47 ^c^	82.81 ^bc^	83.60 ^b^	87.75 ^a^	0.73	0.001
Histidine	66.53 ^c^	69.73 ^bc^	75.85 ^b^	79.24 ^a^	1.51	0.001
Isoleucine	71.92 ^c^	76.84 ^bc^	80.22 ^b^	82.39 ^a^	1.13	0.001
Leucine	83.46	83.19	82.06	85.50	0.49	0.080
Lysine	83.54 ^b^	81.65 ^b^	83.28 ^b^	86.96 ^a^	0.56	0.001
Methionine	84.48 ^a^	76.31 ^b^	72.32 ^b^	84.61 ^a^	1.44	0.001
Phenylalanine	77.20	78.65	77.43	79.49	0.53	0.418
Threonine	77.39	71.92	68.13	74.60	1.63	0.229
Valine	79.54 ^b^	79.75 ^b^	80.88 ^ab^	83.64 ^a^	0.59	0.029
Tryptophan	67.25	73.49	73.93	76.43	1.48	0.148
Glycine	71.71 ^b^	70.66 ^b^	71.38 ^b^	75.99 ^a^	0.67	0.005
Total	79.56 ^b^	79.07 ^b^	79.36 ^b^	83.33 ^a^	0.55	0.004
**Non-essential amino acids**						
Alanine	78.64 ^b^	79.96 ^ab^	79.70 ^b^	83.20 ^a^	0.56	0.008
Aspartic acid	78.79 ^b^	80.03 ^b^	81.20 ^b^	85.82 ^a^	0.80	0.001
Cysteine	67.22 ^ab^	64.86 ^ab^	64.66 ^b^	72.39 ^a^	1.14	0.034
Glutamic acid	78.47 ^b^	79.69 ^b^	78.75 ^b^	83.14 ^a^	0.56	0.001
Proline	73.14 ^b^	74.03 ^ab^	72.01 ^b^	77.43 ^a^	0.64	0.004
Serine	83.02 ^b^	83.82 ^a^	79.82 ^b^	84.94 ^a^	0.61	0.005
Tyrosine	71.99 ^b^	75.12 ^ab^	72.12 ^b^	79.93 ^a^	1.11	0.017
Total	76.82 ^b^	77.74 ^b^	76.89 ^b^	81.65 ^a^	0.58	0.001
Total amino acids	78.60 ^b^	79.01 ^b^	79.36 ^b^	84.48 ^a^	0.69	0.001

Abbreviations: CON, basal diet; T1, partial replacing animal fat with full-fat almonds 1%; T2, partial replacing animal fat with full-fat almonds 2%; T3, partial replacing animal fat with full-fat almonds 4%; SEM, standard error of means. Each value is the mean value of 4 replicates. ^a–c^ Means within column with different superscripts differ significantly (*p* < 0.05).

**Table 9:** Effects of full-fat almonds on blood profiles in broiler chickens.

Items	CON	T1	T2	T3	SEM	*p*-Value
Total Protein (g/dL)	3.60	3.52	3.46	3.49	0.03	0.252
Blood Urea Nitrogen (mg/dL)	1.59	1.75	1.50	1.50	0.09	0.765
Creatinine (mg/dL)	0.172	0.187	0.185	0.175	0.005	0.645
Cholesterol (mg/dL)	186 ^a^	171 ^ab^	154 ^bc^	150 ^c^	4	0.001
Glucose (mg/dL)	104	100	108	113	2	0.171

Abbreviations: CON, basal diet; T1, partial replacing animal fat with full-fat almonds 1%; T2, partial replacing animal fat with full-fat almonds 2%; T3, partial replacing animal fat with full-fat almonds 4%; SEM, standard error of means. Each value is the mean value of 4 replicates. ^a–c^ Means within column with different superscripts differ significantly (*p* < 0.05).

**Table 10:** Effects of full-fat almonds on bacteria counts in the cecal-fecal in broiler chickens.

Items, log CFU/g	CON	T1	T2	T3	SEM	*p*-Value
Cecal						
*Lactobacillus*	8.401	8.427	8.395	8.388	0.011	0.696
*E. coli*	6.225 ^a^	6.153 ^ab^	6.069 ^bc^	6.007 ^c^	0.026	0.002
*Salmonella*	6.085	6.175	6.194	6.034	0.030	0.181
**Fecal**						
*Lactobacillus*	7.969	7.973	7.893	7.744	0.044	0.222
*E. coli*	5.784 ^a^	5.691 ^a^	5.571 ^a^	5.327 ^b^	0.050	0.001
*Salmonella*	5.969	5.886	5.843	5.621	0.051	0.070

Abbreviations: CFU, colony-forming unit; CON, basal diet; T1, partial replacing animal fat with full-fat almonds 1%; T2, partial replacing animal fat with full-fat almonds 2%; T3, partial replacing animal fat with full-fat almonds 4%; SEM, standard error of means. Each value is the mean value of 4 replicates. ^a–c^ Means within column with different superscripts differ significantly (*p* < 0.05).

**Table 11:** Effects of full-fat almonds on the incidence of foot-pad dermatitis in broiler chickens.

Items	CON		T1		T2		T3		SEM	*p*-Value
**Score ^1^**	**N**	**%**	**N**	**%**	**N**	**%**	**N**	**%**	-	-
0	18	37.5	17	35.4	19	39.6	16	33.3		
1	26	54.2	21	43.8	15	31.3	12	25.0		
2	4	8.3	9	18.7	10	20.8	13	27.1		
3	0	0.0	1	2.1	4	8.3	7	14.6		
Total	48	100.0	48	100.0	48	100.0	48	100.0		
Average	0.71 ^b^		0.88 ^ab^		0.98 ^ab^		1.23 ^a^		0.06	0.035

Abbreviations: CON, basal diet; T1, partial replacing animal fat with full-fat almonds 1%; T2, partial replacing animal fat with full-fat almonds 2%; T3, partial replacing animal fat with full-fat almonds 4%; SEM, standard error of means. Each value is the mean value of 4 replicates. ^1^ Lesion score: Lesion score was determined as follows: 0, no lesion; 1, lesion covering less than 25% of the sole of the foot, large area lesion; 2, covering between 25% and 50% of the sole of the foot; 3, more than 50% of the lesion of the plantar. ^a,b^ Means within column with different superscripts differ significantly (*p* < 0.05).

## Data Availability

Data sharing is not applicable to this article.
